# Disentangling the Causal Role of Gut Microbiota in Bacterial Liver Abscess: A Mendelian Randomization Study with Clinical Validation

**DOI:** 10.3390/pathogens14111173

**Published:** 2025-11-18

**Authors:** Jingrun Han, Han Yu, Haocheng Xue, Yifan Lu, Shuang Li, Qingkai Zhang, Jianjun Liu, Dong Shang

**Affiliations:** 1 Department of General Surgery, The First Affiliated Hospital of Dalian Medical University, Dalian 116011, China; wslhjr1999@163.com (J.H.);; 2 Laboratory of Integrative Medicine, The First Affiliated Hospital of Dalian Medical University, Dalian 116011, China; 3 Institute (College) of Integrative Medicine, Dalian Medical University, Dalian 116011, China

**Keywords:** bacterial liver abscess, gut microbiota, bacterial metabolites, 16S rDNA sequencing

## Abstract

Bacterial liver abscess (BLA), accounting for approximately 80% of all liver abscesses, is a severe suppurative infection of the liver. Although gut microbiota dysbiosis has been implicated in BLA pathogenesis, causal evidence remains limited. Here, we integrate Mendelian randomization (MR) and clinical cohort studies to systematically evaluate the causal role of gut microbiota in BLA. Using summary-level genetic data from MiBioGen, GWAS Catalog, and the Pan-UK Biobank, we identified several causal microbial taxa: Coprococcus, Veillonellaceae (including Dialister), and Klebsiella were positively associated with BLA risk, whereas Bacteroides and Bifidobacterium appeared protective. Clinical validation confirmed significant enrichment of Veillonella, Dialister, and Streptococcus in the gut and oral microbiota of BLA patients, contrasting with the predominance of Bacteroides and Bifidobacterium in healthy controls. Klebsiella was the most abundant genus in abscess pus, and gut microbial metabolic profiling revealed marked upregulation of glycolytic pathways in BLA patients. These results indicate that gut dysbiosis exacerbates BLA development through microenvironmental disruption and metabolic reprogramming. Our findings provide mechanistic insights into BLA etiology and suggest microbiota-targeted interventions as promising strategies for prevention and treatment.

## 1. Introduction

Bacterial liver abscess (BLA), also known as pyogenic liver abscess (PLA), is characterized by localized suppurative lesions within the liver tissue resulting from bacterial infection, leading to the accumulation of pus [1]. Despite advances in antimicrobial therapy and interventional management, its annual incidence has steadily risen from 10.83 to 15.45 cases per 100,000 population, with a reported mortality rate of 2% to 19% [2]. The disease often presents with nonspecific symptoms such as fever, abdominal pain, and malaise, which can delay diagnosis and complicate treatment [3,4,5].

There are also many other types of liver abscesses that need to be differentiated from BLA. Amoebic liver abscesses (ALA) caused by Entamoeba histolytica, which are more prevalent in endemic regions and often present with right upper quadrant pain and fever, sometimes accompanied by diarrhea [6]. Fungal liver abscesses occur primarily in immunocompromised patients or those with prolonged hospitalization, intravenous catheter use, or broad-spectrum antibiotic exposure [7]. These diverse etiologies underscore the complexity of liver abscess diagnosis and the importance of identifying the underlying cause to guide appropriate therapy.

With the advancement of research into the pathogenesis, diagnosis, and treatment of liver abscess, an increasing number of studies have revealed a close association between the gut microbiota and the development and progression of this disease. The intestine functions not only as a key organ for digestion and nutrient absorption, but also as a regulator of immune homeostasis and overall health through the balanced composition of gut microbiota [8]. Alterations in the intestinal microbial community may serve as a critical factor contributing to systemic diseases, including liver abscess [9,10,11]. Previous studies have suggested associations between specific bacterial taxa and BLA risk; however, causal relationships remain unestablished due to methodological limitations such as small cohort sizes and an overreliance on correlative analyses. The mechanistic pathways linking gut microbial dysbiosis to BLA pathogenesis thus remain poorly defined.

In this study, we employ an integrated approach combining Mendelian randomization and clinical cohort validation to systematically evaluate causal relationships between gut microbiota composition and BLA risk. By synthesizing genetic, microbial, and clinical datasets, we identify specific bacterial genera and species exerting causal effects on BLA susceptibility, unveiling novel mechanisms underlying disease pathogenesis. These results highlight potential targets for early risk prediction, preventive strategies, and therapeutic interventions, ultimately aiming to improve clinical management and patient outcomes.

## 2. Materials and Methods

### 2.1. Data Source of Exposure and Outcome

We used three publicly available gut microbiota GWAS datasets as the exposure data. (1) Whole-genome genotypes and 16S rRNA fecal microbiome data from 18,340 individuals across 24 cohorts were downloaded from the MiBioGen consortium and used as gut microbiota exposure data [12]. (2) Shotgun metagenomic sequencing data of fecal samples from 7738 individuals in the Dutch Microbiome Project (DMP) were obtained from the GWAS Catalog database. A total of 207 taxonomic units (including 5 phyla, 10 classes, 13 orders, 26 families, 48 genera, and 105 species) and 205 gut microbial metabolic pathways were used as gut microbiota exposure data [13]. (3) Shotgun metagenomic data from 5959 individuals with paired gut microbiome profiles were retrieved from the GWAS Catalog and used as exposure data [14]. Genetic data from 1385 liver abscess cases and 408,402 healthy individuals of European ancestry (Code: 571.8) were obtained from the multi-ethnic Pan-UK Biobank (Pan-UKBB) (https://pan.ukbb.broadinstitute.org/, accessed on 4 August 2024) cohort as outcome data.

### 2.2. The Selection of Instrumental Variables

Exposure GWAS data were retrieved, and SNPs associated with gut microbiota were selected using a significance threshold of *p* < 5 × 10^−6^ to identify suitable instrumental variables (IV). Linkage disequilibrium was removed using a clumping threshold of r^2^ = 0.001 and a window size of 10,000 kb. Weak instruments were excluded based on an F-statistic > 10.

### 2.3. Two-Sample Mendelian Randomization

Horizontal pleiotropy was assessed using MR-Egger regression, while heterogeneity among SNPs was evaluated using Cochran’s Q test. A leave-one-out sensitivity analysis was performed to ensure that the causal estimates were not driven by any single SNP. A total of 1135 SNPs were identified from the MiBioGen consortium, and 2453 and 5260 gut microbiota-associated SNPs were obtained from the GWAS Catalog. Two-sample MR analyses were then conducted by integrating the selected exposure-associated SNPs with outcome data.

MR analyses were performed using multiple methods, including Inverse Variance Weighted (IVW), Weighted Median, Weighted Mode, Simple Mode, and MR-Egger regression. To assess the robustness and consistency of causal estimates, results were visualized using scatter plots, forest plots, and funnel plots.

### 2.4. Clinical Validation

#### 2.4.1. Study Population

The diagnosis of BLA is established through an integrated assessment of clinical manifestations, laboratory findings, and imaging evidence [15,16]. Typical symptoms include fever, chills, and right upper quadrant pain with tenderness. Laboratory tests often reveal leukocytosis with neutrophilia and elevated inflammatory markers (e.g., CRP, PCT). Imaging studies (ultrasound, CT, or MRI) demonstrate hypodense or cystic lesions in the liver, and cultures of pus are performed to isolate the pathogen [5,17].

This study established stringent inclusion and exclusion criteria: Patients with liver abscess were required to (i) adults diagnosed with BLA based on typical clinical features plus imaging evidence (no abscesses at other sites), with confirmation by positive pus culture and/or clinical response to appropriate antibiotics, as per published criteria; (ii) willingness to provide stool and/or oral swabs and, when available, percutaneous drainage pus; (iii) ability to give written informed consent and provide complete clinical data. Exclusion criteria included (i) recent antibiotic/immunosuppressant use (≤4 weeks); (ii) incomplete data; (iii) severe comorbidities/malignancies; (iv) poor compliance/psychiatric conditions; (v) concurrent trial participation. Healthy controls were age-/sex-matched, free of acute infection, and without antibiotic/probiotic exposure within 4 weeks.

#### 2.4.2. Sample Collection

Samples included fecal specimens from healthy individuals and from patients clinically diagnosed with BLA, as well as anal swabs, percutaneous drainage pus, and oral swabs from the patient group. All samples were subjected to 16S rDNA sequencing for microbial profiling.

Oral swabs, fecal samples, and pus aspirates were immediately placed into sterile collection tubes. Samples were temporarily stored at 4 °C and transported under cold-chain conditions to the laboratory within 2–4 h. Upon arrival, samples were aliquoted and stored at −80 °C for long-term preservation to minimize repeated freeze–thaw cycles. Pus samples were obtained by ultrasound-guided percutaneous drainage of liver abscesses.

#### 2.4.3. Institutional Review Board Statement

Clinical samples were collected from the Biobank of the First Affiliated Hospital of Dalian Medical University between November 2024 and January 2025. The study was approved by the Medical Ethics Committee of the First Affiliated Hospital of Dalian Medical University (Ethics Approval No. PJ-KS-KY-2025-75, Approval Date 14 February 2025).

#### 2.4.4. 16S rDNA Sequencing of Gut Microbiota

(i) DNA Extraction: Microbial genomic DNA was extracted from all collected samples using the NEBNext^®^ Ultra™ II DNA Library Prep Kit (Cat. No. E7645B, New England Biolabs, Ipswich, MA, USA), following the manufacturer’s instructions. DNA sequencing was performed on the Illumina NovaSeq 6000 platform (Illumina, San Diego, CA, USA). (ii) PCR Amplification: Bacterial diversity was assessed using primers targeting the 16S rDNA V3~V4 region. All PCR reactions were performed in a 15 µL mixture containing 0.2 µM of each primer, ~10 ng template DNA, and Phusion High-Fidelity PCR Master Mix. PCR products were purified with magnetic beads, pooled in equimolar amounts, and subjected to gel electrophoresis for target band recovery. Sequencing libraries were prepared, quantified (Qubit and qPCR), and sequenced on the Illumina platform. (iii) OTU Clustering and Taxonomic Annotation: Raw sequencing data were processed and analyzed using QIIME2 [18]. Operational taxonomic units (OTUs) were clustered and assigned taxonomy based on the SILVA 138.1 reference database (https://www.arb-silva.de/documentation/release-1381/, accessed on 10 March 2025). Microbial composition was profiled at multiple taxonomic levels, including phylum, class, order, family, genus, and species.

### 2.5. Statistical Methods

All MR analyses were conducted using R software version 4.4.2 with the TwoSampleMR package (version 0.6.6). Causal estimates were expressed as odds ratios (ORs) with corresponding 95% confidence intervals (95% CIs). A *p* < 0.05 was considered statistically significant.

Alpha diversity was evaluated to assess community richness and diversity, with the Chao1 index used to estimate species richness and the Shannon index to measure species diversity. Beta diversity was assessed using both weighted and unweighted UniFrac distances, and a UPGMA (Unweighted Pair Group Method with Arithmetic Mean) clustering tree was generated with the UPGMA.tre function. Differences in microbial composition between groups were tested using permutational multivariate analysis of variance (PERMANOVA). Taxonomic abundance across groups and its associations with clinical features were analyzed using the Wilcoxon rank-sum test. All statistical tests were two-tailed, and a *p* < 0.05 was considered statistically significant.

## 3. Result

### 3.1. MR Analysis Results of Gut Microbiota on BLA

In this study, three publicly available gut microbiome sequencing datasets were utilized. Analysis of the MiBioGen dataset identified 12 microbial taxa significantly associated with the risk of BLA. In the Dutch Microbiome Project (DMP), 8 significant microbial taxa and 10 microbial metabolic pathways were found to be associated with BLA risk. Furthermore, a large-scale metagenomic study involving 5959 individuals with genotype-matched gut microbiome profiles revealed 22 microbial taxa significantly associated with BLA.

**Figure 1:**
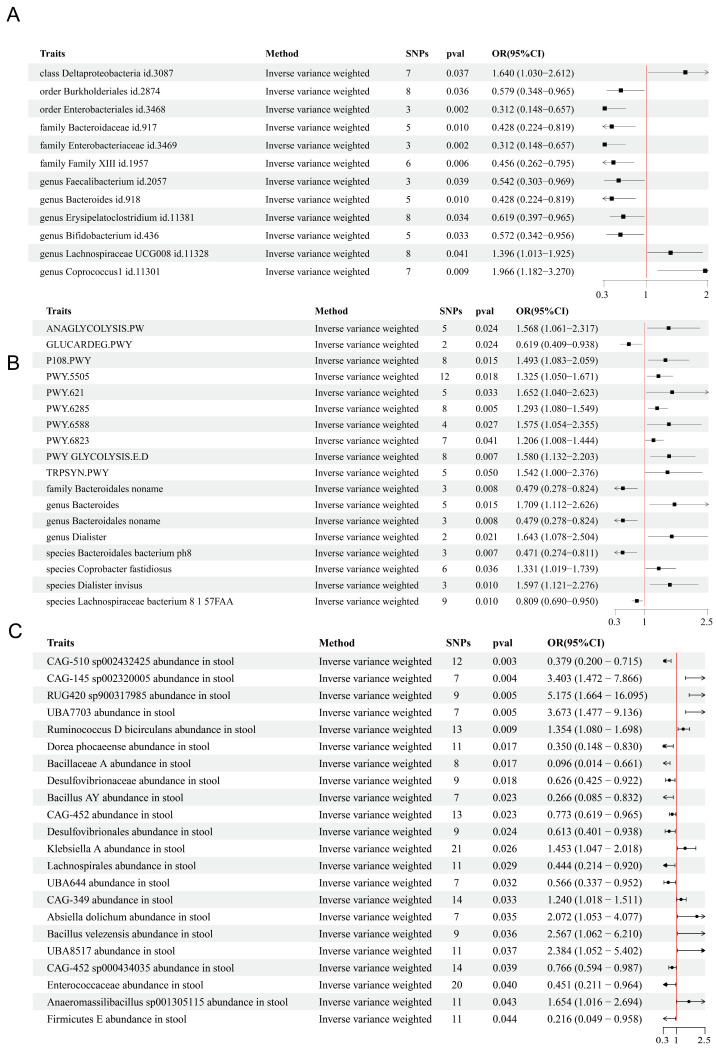
Forest plots of Mendelian randomization estimates for the causal effects of gut microbiota and microbial pathways on liver abscess risk. Each horizontal line represents the 95% CI, and the black spots in the middle of the line indicates the point estimate of the OR. The red line represents the reference for statistical significance: if a horizontal line lies entirely to the right of the red line, it indicates a positive causal effect on the outcome; if it lies to the left, it indicates a negative causal effect. (**A**) Analysis of the first exposure dataset; (**B**) Analysis of the second exposure dataset; (**C**) Analysis of the third exposure dataset.

In the first exposure dataset (Figure 1A), several microbial taxa exhibited significant associations with BLA risk across taxonomic levels. At the class level, *Deltaproteobacteria* (id.3087) was positively associated with BLA. Conversely, at the order level, *Enterobacteriales* (id.3468) and *Burkholderiales* (id.2874) were negatively associated with BLA risk. Similar protective effects were observed at the family level for Family XIII (id.1957), *Enterobacteriaceae* (id.3469), and *Bacteroidaceae* (id.917). At the genus level, *Coprococcus_1* (id.11301) and *Lachnospiraceae_UCG-008* (id.11328) were identified as risk-associated taxa, whereas *Erysipelatoclostridium* (id.11381), *Faecalibacterium* (id.2057), *Bacteroides* (id.918), and *Bifidobacterium* (id.436) were negatively associated with BLA, suggesting a potential protective role.

In the second exposure dataset (Figure 1B), *Bacteroidales*, *Dialister invisus*, *Dialister*, and *Coprobacter fastidiosus* were positively associated with the risk of BLA (*p* < 0.05, OR [95% CI] > 1). In contrast, *Bacteroidales bacterium ph8*, and *Lachnospiraceae bacterium 8_1_57FAA* were negatively associated with BLA risk (*p* < 0.05, OR [95% CI] < 1). It is worth noting that while *Lachnospiraceae UCG-008* and *Coprococcus_1* were identified as risk-increasing taxa for BLA in the first dataset, *Lachnospiraceae bacterium 8_1_57FAA* in the second dataset showed a negative association with BLA risk.

In the third exposure dataset (Figure 1C), multiple taxa demonstrated significant associations with BLA risk. *RUG420 sp900317985*, *UBA7703*, *CAG-145 sp002320005*, *Bacillus velezensis*, *UBA8517* (family level), *Absiella dolichum*, *Anaeromassilibacillus sp001305115*, *Klebsiella A*, *Ruminococcus D*, and *CAG-349* were positively associated with the risk of BLA (*p* < 0.05, OR [95% CI] > 1). Conversely, several taxa—including *Desulfovibrionaceae* (family level), *Desulfovibrionales* (order level), *Enterococcaceae* (family level), *Lachnospirales* (order level), *Dorea phocaeense*, *Bacillus AY*, *Firmicutes E* (phylum level), and *Bacillaceae A* (family level) exhibited negative associations with BLA (*p* < 0.05, OR [95% CI] < 1).

**Figure 2:**
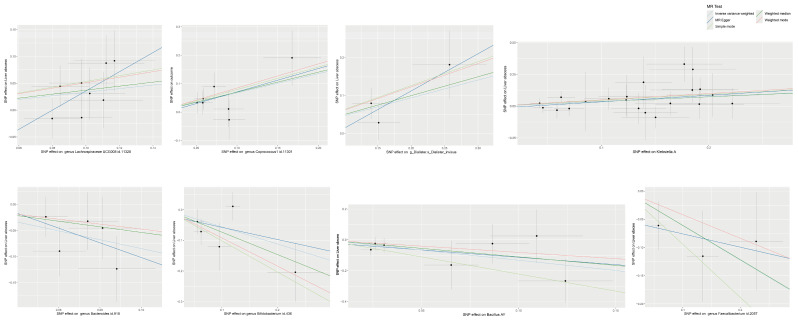
Scatter plots of Mendelian randomization analysis showing SNP effects of gut microbial taxa on the risk of liver abscess. Each black circle represents an individual SNP used as an instrumental variable in the MR analysis.

There exists a strong causal interplay between the gut microbiota and BLA. By organizing the above MR findings and focusing on the family and genus level (Figure 2), we identified several bacterial taxa positively associated with BLA risk, including *Lachnospiraceae*, *Coprococcus*, *Veillonellaceae* (particularly *Dialister*), and *Klebsiella*. In contrast, taxa such as *Bacteroides*, *Bifidobacterium*, *Bacillus*, and *Faecalibacterium* were negatively associated with BLA risk. These findings provide important insights and guidance for subsequent 16S rDNA sequencing analyses of clinical samples.

### 3.2. MR Analysis Results of Gut Microbial Metabolic Pathways on BLA

MR analysis revealed multiple gut microbiota-associated metabolic pathways significantly linked to pyogenic liver abscess (PLA) risk (Figure 1B). Among the positively associated pathways (*p* < 0.05, OR > 1) were key carbohydrate metabolism routes, including glycolysis III (ANAGLYCOLYSIS-PWY), the integrated glycolysis and Entner–Doudoroff superpathway (PWY_GLYCOLYSIS.E.D), sucrose degradation III (PWY.621), pyruvate fermentation to propanoate I (P108.PWY), and pyruvate fermentation to acetone (PWY.6588). Amino acid metabolism pathways such as L-glutamate/L-glutamine biosynthesis (PWY.5505) and L-tryptophan biosynthesis (TRPSYN.PWY) also showed positive associations. Notably, the Escherichia coli fatty acid biosynthesis superpathway (PWY.6285) exhibited the strongest positive signal (*p* = 0.005), alongside molybdopterin cofactor biosynthesis (PWY.6823). In contrast, glucuronate degradation I (GLUCARDEG.PWY) was inversely correlated with PLA risk (*p* < 0.05, OR < 1), suggesting a potential protective role.

In summary, gut microbiota-associated pathways related to carbohydrate, amino acid, and fatty acid metabolism were positively associated with pyogenic liver abscess risk, while glucuronate degradation showed a negative association.

### 3.3. Gut Microbiota Diversity and Community Structure in BLA Patients and Controls

A total of 36 specimens were obtained. Samples were categorized into five groups based on specimen type: fecal samples from BLA patients (FBa group), fecal samples from healthy controls (FBb group), throat swabs (KQ group), and drainage pus (NZ group). All personal identifiers were removed from the specimens prior to analysis. Each sample was anonymized and labeled numerically (e.g., 1, 2, 3, …) to ensure confidentiality and traceability.

**Figure 3:**
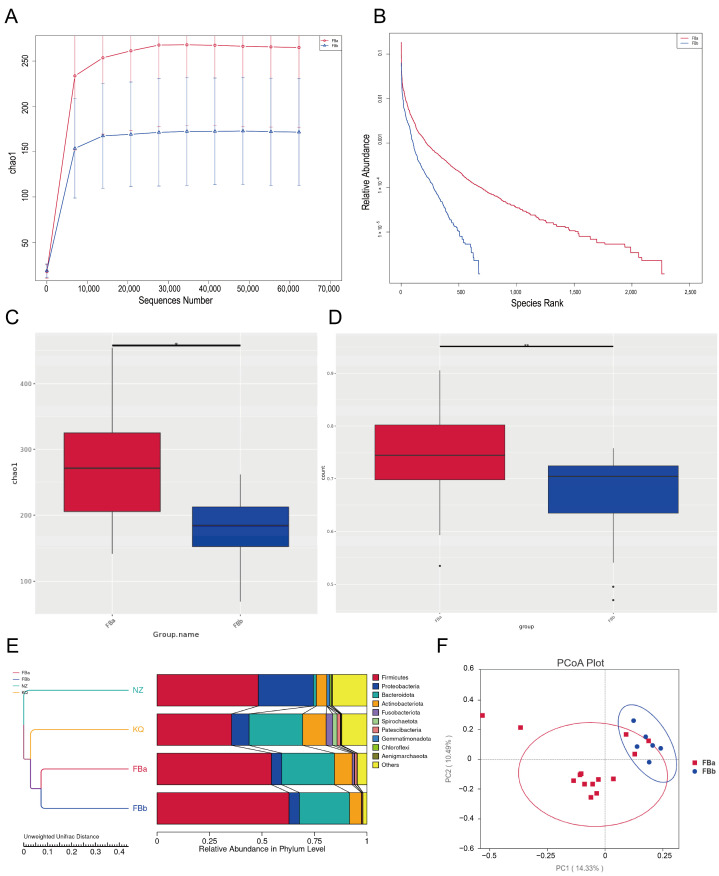
(**A**) Species accumulation curve, with the *x*-axis representing the amount of sequencing data and the *y*-axis representing the corresponding alpha diversity index. (**B**) Alpha diversity rank-abundance curve, where the *x*-axis denotes the rank of feature sequences sorted by abundance, and the *y*-axis shows their relative abundance. (**C**) Alpha diversity (* *p* < 0.05). (**D**) Beta diversity (** *p* < 0.005). (**E**) UPGMA clustering tree at the phylum level showing intergroup similarity; shorter branches denote greater similarity among samples. (**F**) Principal Coordinates Analysis (PCoA).

Alpha diversity analysis primarily focuses on the number of species in a relatively uniform local habitat, and is therefore also referred to as within-habitat diversity. It reflects the richness and diversity of microbial communities within individual samples. To estimate the total number of species in each community sample, the Chao1 index was calculated for each group. A higher Chao1 index indicates greater species richness in the sample. As shown in (Figure 3A,B), the Chao1 index of the FBa group was significantly higher than that of the FBb group, suggesting that the microbial richness in the FBa group was markedly greater than in the FBb group.

Boxplots provide a visual representation of species diversity within groups, including the median, variability, maximum, minimum, and outliers. To identify species with significant differences between groups, a *t*-test was performed. There was a statistically significant difference in gut microbiota richness between the FBa and FBb groups (Figure 3C), indicating that the gut microbiota composition differs between healthy individuals and patients with pyogenic liver abscess.

Building on these within-sample findings, beta diversity analysis was performed to assess inter-sample variation. β diversity analysis compares the microbial community composition between different samples, assessing the degree of variation in microbial composition across groups. There was a significant difference in microbial composition between the FBa and FBb groups (Figure 3D). The UPGMA (Unweighted Pair-group Method with Arithmetic Mean) clustering tree revealed distinct differences in gut microbiota structure between fecal samples from healthy individuals and those from patients with pyogenic liver abscess (Figure 3E). Clustering analysis based on unweighted UniFrac distances revealed that FBa and FBb formed separate branches, indicating significant structural divergence of the gut microbiota between the two groups. In terms of relative abundance, the FBa group was predominantly enriched in *Firmicutes* and *Actinobacteriota*, whereas the FBb group showed higher abundances of *Bacteroidota* and *Proteobacteria*.

Principal Coordinates Analysis (PCoA) based on the Unweighted UniFrac distance was conducted. In this analysis, samples that cluster closer together share more similar microbial community structures, whereas greater distances indicate larger differences. As illustrated in Figure 3F, the gut microbiota structure in BLA patients was markedly altered compared to healthy controls.

### 3.4. Significant Differences in Species Composition Between BLA Patients and Healthy Controls

**Figure 4:**
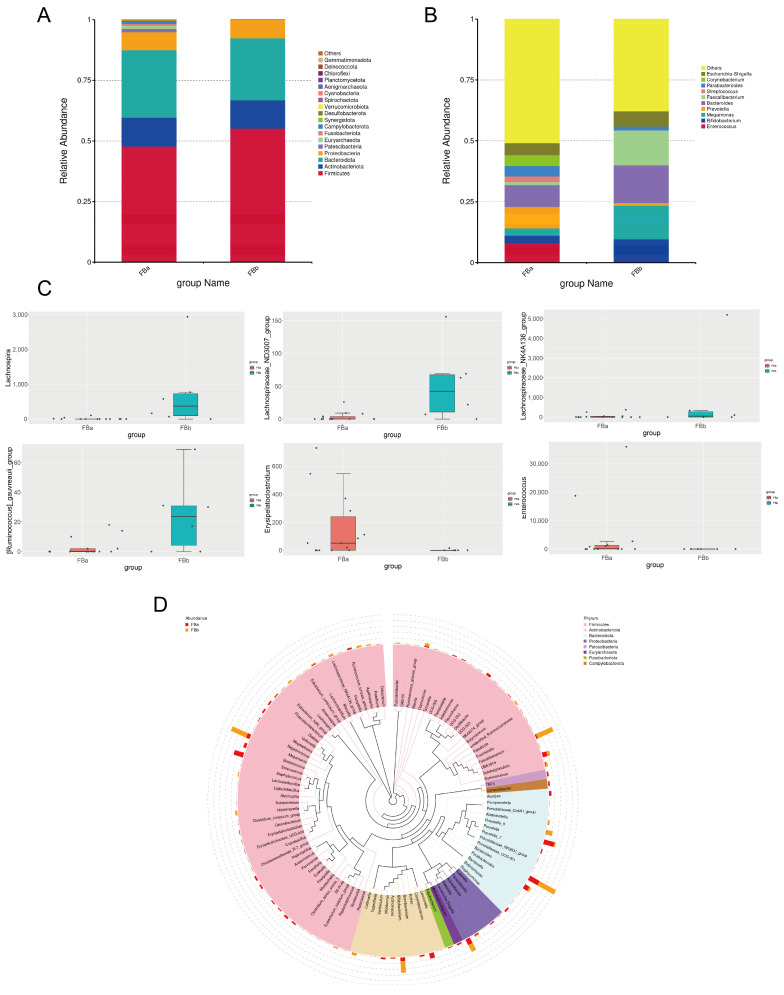
(**A**,**B**) Based on species annotation results at different taxonomic levels, the top 30 genera with the highest relative abundance in each sample or group were selected, with all other genera grouped as “Others.” (**C**) Box plots showing the abundance distribution of significantly different taxa between groups. Each dot represents an individual sample. (**D**) To further explore the phylogenetic relationships at the genus level, representative sequences of the top 100 genera were obtained through multiple sequence alignment, and a phylogenetic tree was constructed.

Through horizontal comparisons, distinct microbial compositions were observed between groups at both the phylum and genus levels (Figure 4A,B). At the phylum level, the relative abundances of *Firmicutes* and *Actinobacteriota* were higher in the FBa group (fecal samples from BLA patients), whereas *Bacteroidota* and *Proteobacteria* were enriched in the FBb group (fecal samples from healthy controls). At the genus level, *Enterococcus*, *Prevotella*, and *Parasutterella* were markedly enriched in the FBa group, while *Bifidobacterium*, *Bacteroides*, *Megamonas*, and *Faecalibacterium* were significantly more abundant in the FBb group.

To identify taxa with significant intergroup differences, we applied the MetagenomeSeq method to perform hypothesis testing on species abundance data, obtaining *p*-values to filter for significantly different genera. Boxplots were generated to visualize the relative abundance distribution of these differential taxa between groups (Figure 4C). The genera with significant differences included *Lachnospira*, *Lachnospiraceae_ND3007_group* and *Lachnospiraceae_NK4A136_group*, all of which were more abundant in healthy fecal samples than in those from BLA patients. These genera are considered potential probiotics and intestinal symbionts, suggesting they may play roles in maintaining gut health and preventing microbiota dysbiosis associated with BLA. In addition, we also found that *[Ruminococcus]_gauvreauii_group* also showed the characteristics of enrichment in the feces of healthy people. Among the bacterial genera with increased abundance in the feces of BLA patients, we found *Erysipelatoclostridium* and *Enterococcus*. Interestingly, *Erysipelatoclostridium* (ID.11381) and *Enterococcaceae* were previously identified as a negative risk factor for BLA in MR analysis.

To further investigate the phylogenetic relationships of genera at the taxonomic level, representative sequences of the top 100 most abundant genera were aligned using multiple sequence alignment, and a phylogenetic tree was constructed (Figure 4D). The results showed that *Dialister*, *Alistipes*, and *Prevotella* were more abundant in the FBa group, highlighting their possible association with disease-specific microbial shifts.

### 3.5. Key Differential Metabolic Pathways in BLA

**Figure 5:**
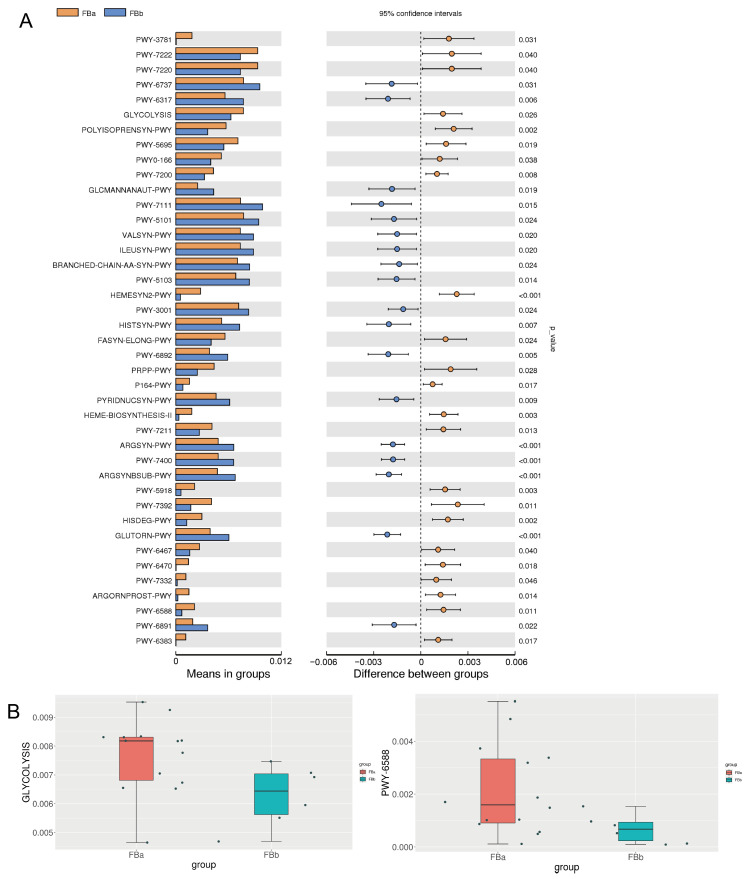
(**A**) *t*-test analysis of microbial metabolic pathways. (**B**) Box plots of significantly different gut microbiota-associated metabolic pathways between groups.

*t*-test analysis was performed to identify differences in microbial metabolic pathways. The glycolysis pathway (GLYCOLYSIS, *p* = 0.026) and the pyruvate fermentation to acetone pathway (PWY.6588, *p* = 0.011) showed significant differences between fecal samples from BLA patients and healthy individuals, as shown in (Figure 5A,B), respectively. These findings are consistent with the MR results presented earlier. Additionally, pathways such as L-arginine biosynthesis I (via L-ornithine) and L-arginine biosynthesis IV (archaea) were found to be more enriched in the gut microbiota of healthy individuals.

### 3.6. Microbial Diversity Differs by Fecal, Oral, and Pus in BLA

To investigate the interactions among fecal, oral, and pus microbiota within BLA patients, we performed a longitudinal comparison based on 16S rDNA sequencing results of the FBa, NZ, and KQ groups.

**Figure 6:**
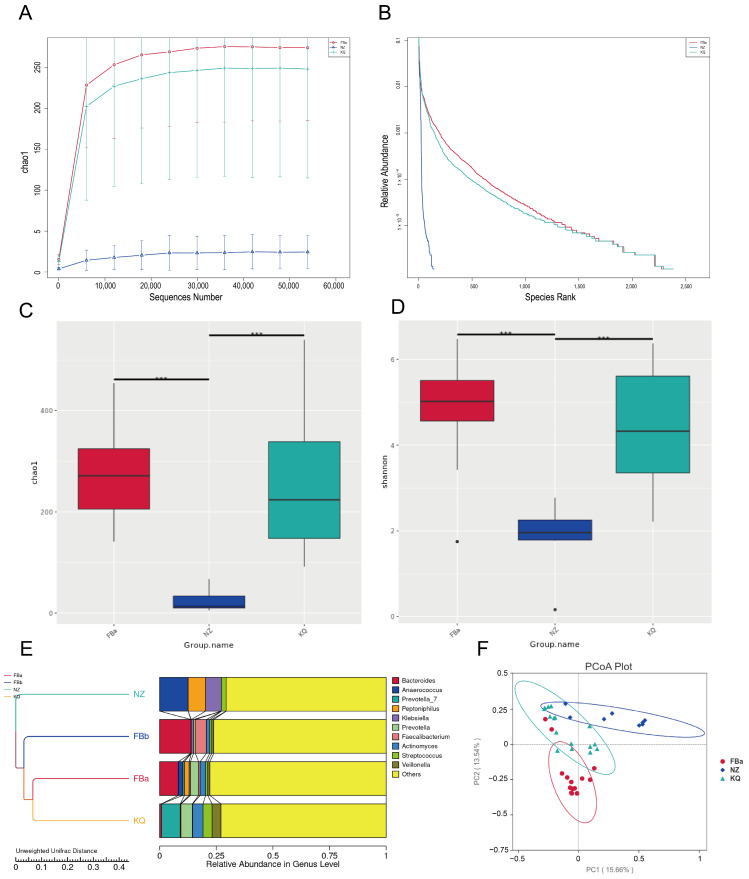
(**A**) Species accumulation curve, where the *x*-axis represents the sequencing depth and the *y*-axis represents the corresponding alpha diversity index. (**B**) Alpha diversity rank-abundance curve, with the *x*-axis showing the rank of feature sequences sorted by abundance and the *y*-axis indicating their relative abundance. (**C**) Alpha diversity analysis (*** *p* < 0.01). (**D**) Beta diversity analysis (*** *p* < 0.01). (**E**) Genus-level UPGMA clustering tree showing intergroup similarity (shorter branches indicate higher similarity); left panel displays the tree, right panel shows taxa relative abundances. (**F**) Principal Coordinate Analysis (PCoA); samples with similar community structures tend to cluster together, while those with distinct communities are positioned farther apart. Each point represents a sample, and the surrounding circle indicates the 95% confidence ellipse for the corresponding group.

As shown in (Figure 6A,B), the Chao1 index in the FBa and KQ groups was significantly higher than that in the NZ group, suggesting that the fecal and oral microbiota of BLA patients exhibited greater species richness and diversity compared to the abscess microbiota. The microbial community in abscess samples appeared to be less diverse and more dominated by a limited number of pathogenic species.

Alpha diversity indices were compared between groups using the Kruskal–Wallis rank sum test. In addition to the Chao1 index, we also calculated the Shannon index, which accounts for both species richness (the number of taxa) and relative abundance (the proportion of each taxon within a sample), serving as a comprehensive indicator of community structure. A higher Shannon index reflects greater community diversity and more even species distribution. As shown in (Figure 6C,D), both the Chao1 and Shannon indices of the NZ group differed significantly from those of the FBa and KQ groups, indicating that the microbial abundance and diversity in abscess samples were markedly different from those in fecal and oral samples of BLA patients.

The UPGMA clustering tree revealed that abscess (NZ group) microbiota formed a distinct branch. The NZ group samples were predominantly composed of Proteobacteria. Genera such as *Anaerococcus*, *Peptoniphilus*, and *Klebsiella* showed higher abundances in the NZ group (Figure 6E). In oral samples, *Prevotella* was dominant, with relatively high abundances of *Streptococcus*, *Veillonella*, and *Actinomyces*.

We also PCoA based on unweighted UniFrac distances, visualizing the results using the principal coordinate axes with the highest contribution. The NZ and FBa groups exhibited a large spatial separation, while the KQ group showed partial clustering with the NZ group (Figure 6F).

**Figure 7:**
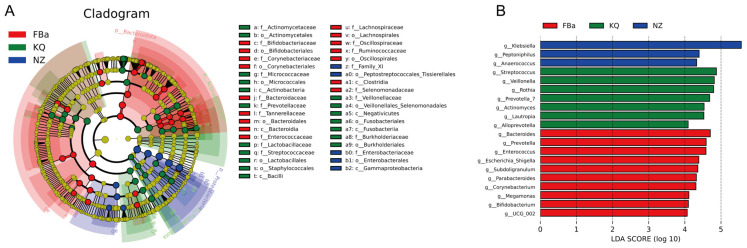
(**A**) Phylogenetic cladogram. The red areas (FBa, BLA patient fecal) are concentrated in certain phyla/classes/orders, indicating that these bacteria are significantly enriched in BLA fecal. The green (KQ, oral cavity) and blue (NZ, abscess) areas show the enrichment of bacterial communities unique to oral and abscess samples, respectively. Species with no significant differences were uniformly colored yellow. (**B**) LDA score distribution bar chart.

LEfSe (Linear Discriminant Analysis Effect Size) is a high-dimensional biomarker discovery tool designed for comparing two or more groups [19]. It emphasizes both statistical significance and biological relevance to identify biomarkers with differential abundance and their associated taxa. LEfSe results include an LDA score distribution histogram and a phylogenetic cladogram (Figure 7). In the abscess samples of BLA patients (NZ group), *Klebsiella* was the predominant genus, followed by *Streptococcus*. Notably, *Streptococcus* was detected in both fecal and oral samples of BLA patients but was absent or present at negligible levels in the feces of healthy controls.

## 4. Discussion

As understanding of BLA pathogenesis and diagnostics advances, evidence increasingly links gut microbiota to disease onset and progression. Alterations in the gut microbial community may serve as a key factor in the development of systemic diseases such as BLA. The gut microbiota comprises a vast array of microorganisms, including bacteria, fungi, viruses, and archaea, among which bacteria are the most abundant in the intestinal tract [20]. Our MR analysis provides genetic evidence supporting a causal relationship between gut microbiota and BLA, in line with previous reports linking microbial dysbiosis to hepatobiliary infections. The integration of microbiome sequencing further underscores the contribution of specific bacterial taxa to disease risk.

Through MR analysis, our study identified specific gut microbial taxa that may influence the risk of BLA. Taxa showing a positive causal association with BLA included *Klebsiella, Lachnospiraceae*, *Coprococcus*, *Veillonellaceae*, and *Dialister*. In contrast, taxa such as *Bacteroides*, *Bifidobacterium*, *Bacillus*, and *Faecalibacterium* exhibited a negative causal association, suggesting a potential protective role against BLA development.

*Klebsiella*, *Escherichia*, *Enterobacter*, and *Streptococcus* are the predominant pathogens responsible for BLA in China [21]. Hypervirulent *Klebsiella* pneumoniae (hvKP) can cause severe extrahepatic infections, including endophthalmitis, meningitis, and necrotizing fasciitis [22]. Clinical studies report high prevalence of hvKP, particularly serotype K1/ST23, among BLA cases, often with multidrug resistance [2,23]. In our study, Klebsiella abundance was not significantly different in feces between BLA patients and controls but was markedly enriched in abscess pus, and MR analysis indicated a risk association with BLA.

*Streptococcus*, widely present in the oral cavity, gastrointestinal tract, and respiratory tract, includes both commensal and pathogenic species. It has been linked to liver injury severity in alcoholic liver disease [24], and its abundance, along with *Veillonella*, correlates positively with serum bile acid levels [25], indicating a connection between hepatic dysfunction and gut microbiota composition. In our study, *Streptococcus* was enriched in both oral and fecal samples of BLA patients, with significantly higher fecal abundance compared to controls, and was also frequently isolated from abscess specimens, including *Streptococcus viridans*. These findings suggest that *Streptococcus* proliferation may contribute to BLA pathogenesis.

*Veillonellaceae*, a family within *Firmicutes*, is broadly distributed across the oral, gastrointestinal, genitourinary, and respiratory tracts and has been implicated in various liver diseases, including autoimmune hepatitis [26], NAFLD [27], and intrahepatic cholangiocarcinoma [28]. In our study, MR analysis revealed a positive association between the genus *Dialister* (a member of the *Veillonellaceae* family) and the risk of BLA. Microbiome sequencing further showed elevated abundances of *Veillonella* and *Dialister* in the oral cavities of BLA patients. These findings suggest that oral Veillonellaceae may act as opportunistic pathogens contributing to BLA pathogenesis.

In contrast, *Lachnospiraceae*, as well as a family within *Firmicutes*, plays a crucial role in gut homeostasis by producing short-chain fatty acids (SCFAs), regulating immunity, and preserving intestinal barrier function [29,30,31,32]. It has also been reported to enhance antitumor immune surveillance and mitigate acute liver injury when enriched through fecal microbiota transplantation [33]. In our study, MR analysis indicated that *Lachnospiraceae_UCG-008* and *Coprococcus_1* were associated with an increased risk of BLA, while some subgroups showed potential protective effects. Consistently, microbiome sequencing showed that *Lachnospiraceae* abundance was significantly reduced in BLA patients compared to healthy controls.

It is noteworthy that not all microbial taxa exert detrimental effects; some genera have been reported to be associated with protective functions. *Bacteroides*, as predominant gut commensals, exert protective effects on liver function via gut–liver axis modulation, including metabolic regulation and SCFA production [34,35,36]. However, some bacterial genera that are commonly considered probiotics, such as *Lactobacillus* [37], can also cause hepatic abscesses. Although no positive results were obtained in this study, this phenomenon still warrants further investigation.

Microbiota-associated metabolic pathways may also contribute to BLA pathogenesis. Glycolysis, the anaerobic conversion of glucose to pyruvate, can be modulated by the gut microbiota; for instance, microbial suppression of glycolysis inhibits lymphopoiesis in calorie-restricted mice [38]. Pyruvate, as a microbial metabolite, activates GPR31 to promote transepithelial dendrite formation in intestinal dendritic cells and enhance immune responses [39]. Moreover, *Prevotella* has been shown experimentally to influence blood glucose levels [40]. Interestingly, although no causal link between *Prevotella* and BLA was detected in MR analysis, 16S rDNA sequencing revealed significant enrichment of *Prevotella* in abscess pus and fecal samples from BLA patients, suggesting its potential involvement in glycemic regulation in the context of BLA. Further studies are warranted to clarify whether *Prevotella* acts as a contributor or a consequence of BLA pathogenesis. Gut microbiota can modulate host immunity and metabolism through their metabolites, potentially influencing BLA onset and progression. Dysbiosis may disrupt microbial metabolites, including fatty acids, amino acids, and bile acids, thereby increasing BLA risk.

Despite these findings, several limitations should be acknowledged. First, the follow-up duration was relatively short, which may limit the assessment of long-term outcomes and recurrence. Second, this study was conducted in a single-country setting, potentially restricting the generalizability of our results to populations with different epidemiological backgrounds. Third, the lack of external validation in independent cohorts limits the robustness and broader applicability of our predictive models. Future studies are needed to clarify how microbial imbalances and their metabolic products drive BLA pathogenesis, which may inform novel strategies for early diagnosis, prevention, and treatment. From a clinical perspective, this study provides a novel framework linking host genetics and gut microbiota to the risk of BLA. Such insights may contribute to developing microbiota-based risk stratification tools and individualized therapeutic strategies for patients susceptible to pyogenic liver abscess.

## 5. Conclusions

The gut microbiota is a key potential source of infection in BLA. *Klebsiella* was identified as a risk-associated genus, showing the highest detection rate in pus microbiota sequencing. *Veillonella*, *Dialister*, and *Streptococcus* were enriched in both the fecal and oral samples of BLA patients. The glycolysis pathway, linked to gut microbiota, was positively associated with BLA risk and elevated in the feces of patients, suggesting its potential role in disease pathogenesis. In contrast, *Bacteroides* and *Bifidobacterium*, which were negatively associated with BLA risk, were reduced in the gut microbiota, indicating possible targets for prevention and treatment.

By comparing the gut microbiota between healthy individuals and BLA patients, this study provides new perspectives on BLA pathogenesis and a theoretical basis for developing future diagnostic and therapeutic strategies.

## Data Availability

The data that support the findings of this study are available from the corresponding author upon reasonable request.
